# Accessory parameters in conformal mapping: exploiting the isomonodromic tau function for Painlevé VI

**DOI:** 10.1098/rspa.2018.0080

**Published:** 2018-08-29

**Authors:** Tiago Anselmo, Rhodri Nelson, Bruno Carneiro da Cunha, Darren G. Crowdy

**Affiliations:** 1 Departamento de Física, Universidade Federal de Pernambuco, 50670-901 Recife, Brazil; 2 Department of Mathematics, Imperial College London, 180 Queen's Gate, London SW7 2AZ, UK

**Keywords:** conformal mapping, accessory parameters, isomonodromy, tau function

## Abstract

We present a novel method to solve the accessory parameter problem arising in constructing conformal maps from a canonical simply connected planar region to the interior of a circular arc quadrilateral. The Schwarz–Christoffel accessory parameter problem, relevant when all sides have zero curvature, is also captured within our approach. The method exploits the isomonodromic tau function associated with the Painlevé VI equation. Recently, these tau functions have been shown to be related to certain correlation functions in conformal field theory and asymptotic expansions have been given in terms of tuples of the Young diagrams. After showing how to extract the monodromy data associated with the target domain, we show how a numerical approach based on the known asymptotic expansions can be used to solve the conformal mapping accessory parameter problem. The viability of this new method is demonstrated by explicit examples and we discuss its extension to circular arc polygons with more than four sides.

## 1. Introduction

The theory of conformal mapping has a long history with perennial interest in it due to its role as an invaluable tool in applied contexts such as fluid dynamics [1,2], solid mechanics [3,4] and in the study of free boundary problems in porous media [5].

The Riemann mapping theorem guarantees that any simply connected planar domain is conformally equivalent to an upper-half plane. However, the theorem is non-constructive and there is a vast literature on analytical and numerical constructions of conformal mappings in various contexts [6].

A famous result is the formula for the conformal mapping from a simply connected canonical domain to a simply connected polygon; this is given by the Schwarz–Christoffel (SC) formula [6,7]. For a triangle, the parameters appearing in the mapping formula can all be determined directly from the vertex positions. By contrast, for polygons with four or more edges the relevant parameters are not all determined and a set of the so-called accessory parameters must be found that cannot be related, at least in any obvious way, to the geometry of the polygon. Research on this class of mappings continues, with the extension of the SC formula to multiply connected polygons found only quite recently [8,9].

Polygons are a special case of more general circular arc polygons, and the theory here is also well developed in the simply connected case [7], with recent work again providing the multiply connected extensions [10,11]. For circular arc polygons an explicit formula (up to accessory parameters) for the conformal mapping from an upper half plane (UHP), say, cannot in general be written down, although an explicit expression for its Schwarzian derivative is available (again, up to accessory parameters). This leads [6,7,12] to a third-order nonlinear equation for the mapping that can be linearized; as a result, the required mapping is given as a bilinear combination of two independent solutions of a second-order Fuchsian differential equation. Such mappings to circular-arc polygons arise frequently in applications. The connection between Fuchsian differential equations, conformal mappings and solutions of free boundary problems in groundwater flows has been exploited to great effect by Polubarinova-Kochina [5], for example.

The principal difficulty in all these problems is solving for the accessory parameters. For SC mappings to simply connected domains, the theory is, by now, well developed [13] with powerful software available for general use (see discussion of the SCToolbox in [13]). Numerical solution of the accessory parameter problem for circular arc polygons has been explored [14,15], but there are no general-use codes and many numerical issues in solving the accessory parameter problem in the general case still exist. The phenomenon of ‘crowding’, in which very small regions of the preimage curve are mapped to extensive regions of the target curve, are a ubiquitous and significant source of numerical difficulty for most computational approaches.

The purpose of this paper is to offer a novel mathematical perspective on the accessory parameter problem in constructing conformal mappings to circular arc polygons. It is not merely of theoretical interest: it has the advantage that numerical implementation of it appears not to suffer from the aforementioned crowding problems. We focus here on a pedagogical introduction to these ideas in the case of circular arc quadrilaterals (four sides). The approach is fundamentally interdisciplinary with ideas imported from the theory of isomonodromic deformations of Fuchsian differential equations and use of an associated tau function. We are not aware of any previous application of such ideas to the construction of circular arc polygons.

Now for a few conventions: the conformal (or ‘uniformizing’) map *z* = *f*(*w*) from a canonical simply connected domain in the complex plane, say, the UHP, to a domain bounded by a series of circular arcs and/or straight lines—a ‘polycircular arc domain’—with *n* vertices satisfies the Schwarzian differential equation [12]

**Figure 1.**
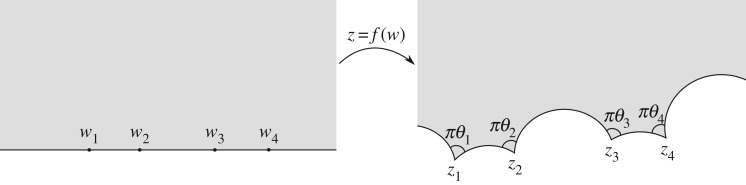
Conformal mapping from the UHP to the interior of a polycircular arc domain, with *z*_*i*_ = *f*(*w*_*i*_).

1.1 $$\{\;f(w),w\}:={\left(\frac{f^{\prime \prime }}{f^{\prime }}\right)}^{\prime }-\frac{1}{2}{\left(\frac{f^{\prime \prime }}{f^{\prime }}\right)}^{2}=\sum_{i=1}^{n}\left[\frac{1-\theta_{i}^{2}}{2{\left(w-w_{i}\right)}^{2}}+\frac{\beta_{i}}{w-w_{i}}\right],$$

where *θ*_*i*_*π* are the interior angles at each vertex *z*_*i*_ = *f*(*w*_*i*_) in the target domain *D*, *w*_*i*_ are the positions of the pre-vertices, and *β*_*i*_ are called the accessory parameters (figure 1).

The study of the uniformizing map can be related to the theory of Fuchsian equations by considering that a solution of (1.1) is written as $f(w)={\tilde{y}}_{1}(w)/{\tilde{y}}_{2}(w)$, where ${\tilde{y}}_{1}(w)$ and ${\tilde{y}}_{2}(w)$ are two linearly independent solutions of the second-order equation [6]

1.2 $${\tilde{y}}^{\prime \prime }(w)+\sum_{i=1}^{n}\left[\frac{1-\theta_{i}^{2}}{4{\left(w-w_{i}\right)}^{2}}+\frac{\beta_{i}}{2(w-w_{i})}\right]\tilde{y}(w)=0,$$

a generic Fuchsian equation with *n* regular singular points at the pre-vertices *w* = *w*_*i*_. Regularity of *f*(*w*) as w→∞ in (1.1), and therefore of ${\tilde{y}}_{1,2}(w)$ as w→∞ in (1.2), requires the algebraic equations

1.3 $$\sum_{i=1}^{n}\beta_{i}=\sum_{i=1}^{n}(2w_{i}\beta_{i}+1-\theta_{i}^{2})=\sum_{i=1}^{n}(\beta_{i}w_{i}^{2}+w_{i}(1-\theta_{i}^{2}))=0,$$

leaving us with *n* − 3 independent *β*_*i*_'s. By the same token, one can use the invariance of (1.1) by Möbius transformations to fix three of the pre-vertices *w*_*i*_. This leaves the 4-vertex case as the first non-trivial one. This is the case we will consider in the paper.

Let the pre-vertices be located at *w*_*i*_ = 0, *t*_0_, 1, ∞ and the internal angles at the corresponding vertices be *θ*_*i*_*π*, with *θ*_*i*_∈{*θ*_0_, *θ*_*t*_0__, *θ*_1_, *θ*_∞_0__}. For the purpose of embedding this equation in a Fuchsian-like system in §3, it is very convenient to transform the Heun equation to its so-called canonical form:

1.4 $$y^{\prime \prime }(w)+\left(\frac{1-\theta_{0}}{w}+\frac{1-\theta_{t_{0}}}{w-t_{0}}+\frac{1-\theta_{1}}{w-1}\right)y^{\prime }(w)+\left[\frac{q_{+}q_{-}}{w(w-1)}-\frac{t_{0}(t_{0}-1)K_{0}}{w(w-1)(w-t_{0})}\right]y(w)=0,$$

where $q_{\pm }=1-\frac{1}{2}(\theta_{0}+\theta_{t_{0}}+\theta_{1}\pm \theta_{\infty_{0}})$ and

1.5 $$K_{0}=-\frac{1}{2}\left[\beta_{t_{0}}+\sum_{k\neq t_{0}}\frac{\left(1-\theta_{t_{0}})(1-\theta_{k}\right)}{t_{0}-w_{k}}\right].$$

We note that $\tilde{y}(w)$ and *y*(*w*) are related by a ‘s-holomorphic transformation’: $\tilde{y}(w)=\phi (w)y(w)$, for *ϕ*(*w*) = *w*^−*θ*_0_/2^(*w* − 1)^−*θ*_1_/2^(*w* − *t*_0_)^−*θ*_*t*_0__/2^, and hence *f*(*w*) = *y*_1_(*w*)/*y*_2_(*w*) can be computed with a pair of solutions from either (1.4) or (1.2). Hereafter, we generically refer to both *t*_0_ and *K*_0_ in equation (1.4) as accessory parameters.

As we will see in the following, the problem we address is the original version of the Riemann–Hilbert problem (RHp): how to find the accessory parameters in (1.4), or equivalently (1.2), given the monodromy data of the solutions. As will be shown in §2, the monodromy data are tied to the geometric properties of the domain. This can be seen from either the parallel transport along the perimeter, or by the explicit construction outlined in §2. For (1.4) or, equivalently (1.2), the monodromy data comprise four monodromy matrices *M*_*i*_ each associated with a singular point. The matrix *M*_*i*_ encodes the result of analytic continuation around the singular point *w*_*i*_ of a particular solution to (1.4). Given that there are two linearly independent solutions to this equation, the matrices are defined up to conjugation, meaning a change of basis of solutions. A convenient set of invariant coordinates to the space of monodromy data are the trace coordinates

$$\rho =\{\mathrm{Tr}\;M_{i},\mathrm{Tr}\;M_{i}M_{j},\mathrm{Tr}\;M_{i}M_{j}M_{k},\ldots \}.$$

The RHp that we tackle in this paper is to find the accessory parameters of ODEs such as (1.4), or the associated matricial system we will define below, from the monodromy data *ρ*.

The most convenient way to deal with the RHp is to cast equation (1.4) into a matricial system of first order ∂_*w*_*Φ*(*w*) = *A*(*w*)*Φ*(*w*)—see (3.1). Given the number of parameters of the partial fraction expansion of *A*(*w*), one realizes a family of equations with the same monodromy data. This isomonodromy family has its parameters related by the Schlesinger equations (3.6). It is a well-established fact that the second-order equation derived from the generic matricial system can have extra singularities—see (3.3). These are apparent singularities, meaning that the monodromy matrices associated with them are identity matrices. The Schlesinger equations induce a Hamiltonian dynamics on the apparent singularities (3.5). The solutions of these Hamiltonian equations possess the Painlevé property: their movable singularities (that is, those dependent on initial conditions) are poles. Functions with the Painlevé property were recognized at the end of the nineteenth century to constitute an important subclass of functions defined by solutions of ordinary differential equations. The four-singularities case corresponds to the Painlevé VI transcendent.

Despite having the Hamiltonian structure outlined in the beginning of the twentieth century, the dependence of the isomonodromic Hamiltonian flow on the monodromy data was mysterious until a series of papers from Jimbo, Miwa, Ueno and collaborators in around 1980 [16,17]. Motivated by applications to integrable models, distribution functions of statistical mechanics and random matrix models, they introduced the isomonodromic tau function, which is related by a logarithmic derivative to the value of the Hamiltonian under the isomonodromic flow, and showed that it satisfies the Painlevé property.

Applications of the Jimbo–Miwa–Ueno tau functions, closer in spirit to the topic of this paper, include the connection problem for the Heun differential equation, which was used to study scattering of scalar fields in black hole backgrounds [18,19] as well as the quantization of the Rabi model in quantum optics [20].

Another connection to our work is the observation that the tau function for the Painlevé VI transcendent coincides with the Fourier transform of a particular 4-point Virasoro conformal block for *c* = 1, relating the Painlevé transcendents with the representation theory of the Virasoro algebra, and hence to (quantum) Liouville field theory. The latter has been studied for a long time due to its ties to string theory, and, by the AGT conjecture [21], is given by certain partition functions of supersymmetric gauge theories. The AGT conjecture [21], proved in [22], allows for an exact expansion of the Virasoro conformal blocks in terms of sums of tuples of Young diagrams, which we record in appendix A. By the observation made in [23], the same conformal block applies to the semi-classical limit of Liouville field theory, which has been related to the existence of the accessory parameters for the classical uniformization problem in [24]. In this paper, we give a constructive procedure for this calculation.

The paper is structured as follows. In §2, we describe the construction of the monodromy matrices and the monodromy data for generic *n*-vertex polycircular arc domains, even though we will focus here on the *n* = 4 case. Section 3 outlines the isomonodromy method used to calculate the accessory parameters *t*_0_ and *K*_0_. After some considerations in §4 we present some examples in §5. We close with some remarks and conjectures.

## 2. Finding the monodromies

Let us start with two linearly independent solutions *y*_1_(*w*) and *y*_2_(*w*) of (1.4), arranged as a (row) vector *Y* (*w*) = [*y*_1_(*w*) *y*_2_(*w*)]. Analytic continuation along a closed loop *γ*_*i*_ around a singular point *w*_*i*_ brings *Y* (*w*) to

$$Y_{\gamma_{i}}(w)\equiv Y(e^{2\pi i}(w-w_{i})+w_{i})=Y(w)M_{i},$$

where *M*_*i*_, called a monodromy matrix, implements a linear combination between the elements of *Y* (*w*) due to the existence of a branch point at *w*_*i*_ as a consequence of *w*_*i*_ being a regular singular point of (1.4). The elements of *M*_*j*_ will depend both on the parameters of the equation as well as the choice of solutions. By picking a different set of linearly dependent solutions one constructs a new vector $\tilde{Y}(w)$ related to the previous one by Ỹ(*w*) = *Y* (*w*)*F*, where F∈GL(2,C). This change implies the transformation in *M*_*i*_:

$${\tilde{M}}_{i}=F^{-1}M_{i}F.$$

Since the particular form of *F* depends on this choice, we see that the set of monodromy matrices {*M*_*i*_} is defined up to conjugation. A similar transformation happens if we deform *γ*_*i*_ inside the same homotopy class, because then the difference between the contours would be a closed path inside which both functions are analytic. Therefore, for our particular example, *M*_*i*_, *i* = 0, *t*, 1, ∞, generate a conjugacy class which is a representation of the fundamental homotopy group of $P^{1}/\{0,1,t,\infty \}$. This is so because of composition: the monodromy matrix associated with two independent contours *γ*_*i*_ and *γ*_*j*_ is *M*_*j*_*M*_*i*_. Notice that, since we will later view *t*_0_ as a time-like variable parametrizing the isomonodromic deformations, we are now denoting it simply by *t*.

Given that the contour encompassing all singular points is contractible, we have the following relation for the set of monodromy matrices

2.1 $$M_{\infty }M_{1}M_{t}M_{0}=\text{𝟙}.$$

Also, because of the equivalence of sets of monodromy matrices by overall conjugation described above, it is desirable to associate with the set of monodromy matrices the invariant parameters:

2.2 $$2\cos\pi \alpha_{i}=\mathrm{Tr}\;M_{i},\;2\cos\pi \sigma_{ij}=\mathrm{Tr}\;M_{i}M_{j}.$$

In our problem, we are given the geometrical representation of the domain we set out to uniformize, and this allows us to compute the parameters defined above. Remember that the uniformizing map is given by the ratio of two linearly independent solutions of (1.2), *f*(*w*) = *y*_1_(*w*)/*y*_2_(*w*), which are analytic except at the singular points of their defining equation. Hence, *M*_*i*_ is related to the manner in which *f*(*w*) transforms under an analytic continuation around *w*_*i*_. For our application, the singular points are located at the boundary of the domain, which is the image of the real line $z=f(w=\bar{w}),\bar{z}=\bar{f}(w=\bar{w})$. A convenient description of the boundary is given by the Schwarz funcion $\bar{z}=S(z)$ [25]. We will deal with the case where the boundary consists of a connected sequence of circular arcs or straight lines {*C*_*i*_}, a *polycircular arc domain* for short. On each arc *C*_*i*_, we have

2.3 $$S_{i}(z):=\bar{z}={\bar{z}}_{i}+\frac{r_{i}^{2}}{z-z_{i}}=\frac{{\bar{z}}_{i}z+r_{i}^{2}-|z_{i}{|}^{2}}{z-z_{i}},$$

where *z*_*i*_ is the centre of circle to which *C*_*i*_ belongs, *r*_*i*_ is its radius, and the Schwarz function *S*_*i*_(*z*) and its inverse function are defined on an open set containing a point in the interior of *C*_*i*_. One can use this fact to continue *z* = *f*(*w*) past the real line: for *w* in the lower half plane, $\bar{f}(\bar{w})$ is defined and analytic near the real line and ${\bar{S}}_{i}(\bar{z})={\bar{S}}_{i}(\bar{f(w)})$ agrees with *z* for $w=\bar{w}$. This is the Schwarz reflection principle.

For a circular arc domain, *S*_*i*_(*z*) is given locally as a Möbius transformation such as (2.3). Abusing notation and using the same *S*_*i*_ now to denote a 2 × 2 matrix representing the action of this Möbius transformation, the action of the Schwarz reflection principle on the vector *Y* of solutions is

2.4 $$\bar{Y}(w)=Y(w)S_{i},\;S_{i}=\frac{i}{r_{i}}\left(\begin{array}{cc} {\bar{z}}_{i} & 1\\ r_{i}^{2}-|z_{i}{|}^{2} & -z_{i} \end{array}\right),$$

with the prefactor chosen so that *S*_*i*_ is unimodular, and $S_{i}{\bar{S}}_{i}=\text{𝟙}$. If *γ*_*i*_ is a sufficiently small closed curve containing *z*_*i*_, the monodromy picked by *Y* (*w*) as one follows the curve counterclockwise is

2.5 $$Y_{\gamma_{i}}(w)=Y(w)S_{i+1}{\bar{S}}_{i}$$

as we compose the continuation through *C*_*i*_ and back through *C*_*i*+1_. This establishes the monodromy matrix *M*_*i*_ around *z*_*i*_ explicitly.

The definition $M_{i}=S_{i+1}{\bar{S}}_{i}$, with ${\bar{S}}_{i}S_{i}=\text{𝟙}$, automatically satisfies (2.1). From

2.6 $$M_{i}=\frac{1}{r_{i}r_{i+1}}\left(\begin{array}{cc} z_{i}{\bar{z}}_{i+1}+r_{i}^{2}-|z_{i}{|}^{2} & {\bar{z}}_{i+1}-{\bar{z}}_{i}\\ z_{i}(r_{i+1}^{2}-|z_{i+1}{|}^{2})-z_{i+1}(r_{i}^{2}-|z_{i}{|}^{2}) & {\bar{z}}_{i}z_{i+1}+r_{i+1}^{2}-|z_{i+1}{|}^{2} \end{array}\right)$$

we have

2.7 $$2\cos\pi \alpha_{i}=\mathrm{Tr}\;M_{i}=\frac{z_{i}{\bar{z}}_{i+1}+r_{i}^{2}-|z_{i}{|}^{2}+{\bar{z}}_{i}z_{i+1}+r_{i+1}^{2}-|z_{i+1}{|}^{2}}{r_{i}r_{i+1}}$$

which are related to the internal angles *πθ*_*i*_ between the two segments meeting at *z*_*i*_ by *θ*_*i*_ = 1 − *α*_*i*_. This explicit representation of the monodromy matrices allows us to write all monodromy parameters as

2.8 $$2\cos\pi \theta_{i}=-\mathrm{Tr}\;M_{i},\;2\cos\pi \sigma_{ij}=\mathrm{Tr}\;M_{i}M_{j},$$

where *θ*_*i*_ and *σ*_*ij*_ will be called simple and composite monodromies, respectively.

We will assume for now that at least one radius *r*_*i*_ is finite. A slight technical complication for the method outlined here arises when one considers domains consisting solely of straight lines. For this case, the polycircular arc domain degenerates to a polygon and the uniformizing map is known to be given by the classical SC formula. We will see in §5, however, that we are able to extend the results for generic polycircular arcs to the polygon case by considering a small curvature—large *r*_*i*_—limit of the formulae above.

Not all monodromy parameters are independent: using the Cayley–Hamilton theorem, which for invertible two-dimensional matrices gives

$$M_{i}+M_{i}^{-1}\det M_{i}=\text{𝟙}\mathrm{Tr}\;M_{i},$$

and beginning from (2.1) one can arrive at the Fricke–Jimbo relation:

2.9 $$\begin{array}{rl} J(\theta_{i},\sigma_{ij}) & =p_{0t}p_{1t}p_{01}+p_{01}^{2}+p_{1t}^{2}+p_{01}^{2}+p_{0}^{2}+p_{t}^{2}+p_{1}^{2}+p_{\infty }^{2}+p_{0}p_{t}p_{1}p_{\infty }\\ & \;-(p_{0}p_{t}+p_{1}p_{\infty })p_{0t}-(p_{1}p_{t}+p_{0}p_{\infty })p_{1t}-(p_{0}p_{1}+p_{t}p_{\infty })p_{01}-4=0, \end{array}$$

where *p*_*i*_ = 2cos*πθ*_*i*_ and *p*_*ij*_ = 2cos*πσ*_*ij*_. Therefore, from the set of three composite monodromy parameters *σ*_*ij*_ = {*σ*_0*t*_, *σ*_1*t*_, *σ*_01_}, only two are independent. This is the same number of independent accessory parameters in the differential equation (1.2). The way the monodromy data determine the accessory parameters is best visualized when the Heun equation is written as a Fuchsian system, which is the subject of the next section.

## 3. The Fuchsian system: isomonodromy and the Jimbo–Miwa–Uenotau function

As stated in the introduction, the second order differential equation has in general fewer free parameters than the corresponding monodromy group. These extra parameters can be included in the differential equation if it is cast as a matricial equation. For the Heun equation with four regular singular points we have

3.1 $$\partial_{w}\Phi (w)=A(w)\Phi (w),\;\Phi (w)=\left(\begin{array}{cc} y_{1}(w) & y_{2}(w)\\ u_{1}(w) & u_{2}(w) \end{array}\right),\;A(w)=\frac{A_{0}}{w}+\frac{A_{1}}{w-1}+\frac{A_{t}}{w-t},$$

where the 2 × 2 matrix *A*_*i*_ does not depend on *w* and the residue of *A*(*w*) at infinity implies that *A*_0_ + *A*_*t*_ + *A*_1_ = − *A*_∞_, which can be diagonalized by a suitable transformation Φ(w)→GΦ(w). When all *A*_*i*_'s are traceless we will refer to (3.1) as a Fuchsian system. One can now define the action of monodromy matrices: let *Φ*_*γ*_*i*__(*w*) be the result of an analytic continuation of *Φ*(*w*) along a closed loop *γ*_*i*_ around the singular point *w*_*i*_ of the Fuchsian equation, so that we start with *Φ*(*w*) at an ordinary point and come back to it. Hence,

$$\Phi_{\gamma_{i}}(w)=\Phi (w)M_{i}.$$

Again, choosing a different starting point amounts to picking a monodromy matrix ${\tilde{M}}_{i}=FM_{i}F^{-1}$, for some F∈GL(2,C).

Using (3.1) a second order ODE for *y*_1_(*w*) of the form

3.2 $$y^{\prime \prime }-(\mathrm{Tr}\;A+{\left(\log A_{12}\right)}^{\prime })y^{\prime }+(\det A-A_{11}^{\prime }+A_{11}{\left(\log A_{12}\right)}^{\prime })y=0,$$

is then derived where the subscript 1 in *y*_1_ has been dropped, and *A*_*ij*_ corresponds to the *ij*-entry of *A*(*w*). A similar equation can be found for any other element of *Φ*(*w*). One can further show that *y*_1_(*w*) and *y*_2_(*w*)—as well as *u*_1_(*w*) and *u*_2_(*w*)—are linearly independent when the matrix *Φ*(*w*) is invertible.

Requiring that (3.2) has the same form as (1.4) imposes constraints on the number of free parameters of *A*(*w*). Enforcing that *A*_∞_ is diagonal leads to the assumption that *A*_12_(*w*) vanishes like $O(w^{-2})$ as w→∞. Given the partial fraction expansion of *A*(*w*) we find

$$A_{12}(w)=\frac{k(w-\lambda )}{w(w-1)(w-t)},\;k\in C,$$

so that the off-diagonal element *A*_12_ has a single zero, which we call λ. Some algebra and a comparison with (1.4) reveals that Tr *A*_*i*_ = *θ*_*i*_ and $\det A_{i}=0$. Then, one finds that (3.2) can be written as

3.3 $$\begin{array}{rl} & y^{\prime \prime }+(\frac{1-\theta_{0}}{w}+\frac{1-\theta_{t}}{w-t}+\frac{1-\theta_{1}}{w-1}-\frac{1}{w-\lambda })y^{\prime }\\ & \;+\left(\frac{\kappa_{-}(1+\kappa_{+})}{w(w-1)}-\frac{t(t-1)K}{w(w-t)(w-1)}+\frac{\lambda (\lambda -1)\mu }{w(w-\lambda )(w-1)}\right)y=0, \end{array}$$

where *μ* is the residue of *A*_11_(*w*) at *w* = λ, we chose *A*_∞_ = diag(*κ*_−_, *κ*_+_), with $\kappa_{\pm }=-\frac{1}{2}(\theta_{0}+\theta_{t}+\theta_{1}\pm \theta_{\infty })$, and *K* is given by

3.4 $$K(\lambda ,\mu ,t)=\frac{\lambda (\lambda -t)(\lambda -1)}{t(t-1)}\left[\mu^{2}-\left(\frac{\theta_{0}}{\lambda }+\frac{\theta_{1}}{\lambda -1}+\frac{\theta_{t}-1}{\lambda -t}\right)\mu +\frac{\kappa_{-}(1+\kappa_{+})}{\lambda (\lambda -1)}\right].$$

This relation between *K*, *μ* and λ allows us to show that the singularity of the equation (3.3) at *w* = λ is *apparent*: the indicial exponents at this point are integers (0, 2) and (3.4) guarantees that there is no logarithmic behavior. The monodromy associated with a circuit around *w* = λ is therefore trivial and the corresponding matrix is the identity *M*_λ_ = 𝟙.

The relation between *K*, λ and *μ* also allows us to interpret a change of the singularity position *w* = *t* as inducing a change in the parameters of (3.3) according to the Hamiltonian system

3.5 $$\frac{d\lambda }{dt}=\{K,\lambda \},\;\frac{d\mu }{dt}=\{K,\mu \},\;\{f,g\}=\frac{\partial f}{\partial \mu }\frac{\partial g}{\partial \lambda }-\frac{\partial f}{\partial \lambda }\frac{\partial g}{\partial \mu },$$

where it can be checked that the second order differential equation for λ(*t*) is the Painlevé VI equation (PVI). One can see that this deformation does not change the monodromy parameters by casting them in terms of the matricial system. Let the traceless matrices

$$\hat{A}(w,t)=\frac{{\hat{A}}_{0}}{w}+\frac{{\hat{A}}_{t}}{w-t}+\frac{{\hat{A}}_{1}}{w-1},\;\hat{B}(w,t)=-\frac{{\hat{A}}_{t}}{w-t},$$

where Â_*i*_ does not depend on *w*, satisfy a zero curvature condition

$$\partial_{t}\hat{A}-\partial_{w}\hat{B}+[\hat{A},\hat{B}]=0.$$

In terms of Â_*i*_, this zero curvature condition is equivalent to the Schlesinger equations:

3.6 $$\frac{\partial {\hat{A}}_{0}}{\partial t}=\frac{1}{t}[{\hat{A}}_{t},{\hat{A}}_{0}],\;\frac{\partial {\hat{A}}_{1}}{\partial t}=\frac{1}{t-1}[{\hat{A}}_{t},{\hat{A}}_{1}],\;\frac{\partial {\hat{A}}_{t}}{\partial t}=\frac{1}{t}[{\hat{A}}_{0},{\hat{A}}_{t}]+\frac{1}{t-1}[{\hat{A}}_{1},{\hat{A}}_{t}].$$

Due to the zero curvature condition, and the analyticity of the system, one can prove that the monodromy parameters are preserved by the change in *t*. In particular, the eigenvalues of Â_*i*_, related to the parameters *θ*_*i*_, are conserved under the (isomonodromic) deformation.

For any solution of the Schlesinger equations, the 1-form $\omega =\sum_{i<j}\mathrm{Tr}\;{\hat{A}}_{i}{\hat{A}}_{j}\text{d}\log(w_{i}-w_{j})$ is closed [16]. This allows for the definition of a tau function as $\omega =\text{d}\log\hat{\tau }$. In simpler terms:

3.7 $$\frac{d}{dt}\log\hat{\tau }(t)=\frac{1}{t}\mathrm{Tr}\;{\hat{A}}_{0}{\hat{A}}_{t}+\frac{1}{t-1}\mathrm{Tr}\;{\hat{A}}_{1}{\hat{A}}_{t}.$$

The tau function is related to the parameters of (3.3) by

3.8 $$\frac{d}{dt}\log\hat{\tau }(t)=K+\frac{\theta_{0}\theta_{t}}{t}+\frac{\theta_{1}\theta_{t}}{t-1}-\frac{\kappa_{-}(\lambda -t)}{t(t-1)}-\frac{\lambda (\lambda -1)\mu }{t(t-1)}.$$

Utilizing the Schlesinger equations (3.6), one can show that $d\log\hat{\tau }/dt$ obeys a differential equation: consider the function $\hat{\zeta }(t)$ below and its derivatives

3.9 $$\hat{\zeta }(t):=t(t-1)\frac{d}{dt}\log\hat{\tau }(t),\;{\hat{\zeta }}^{\prime }(t)=\mathrm{Tr}\;{\hat{A}}_{0}{\hat{A}}_{t}+\mathrm{Tr}\;{\hat{A}}_{t}{\hat{A}}_{1},\;{\hat{\zeta }}^{\prime \prime }(t)=\frac{\mathrm{Tr}[{\hat{A}}_{0},{\hat{A}}_{t}]{\hat{A}}_{1}}{t(1-t)}.$$

Any triple of traceless 2 × 2 matrices obeys

3.10 $${\left(\mathrm{Tr}[{\hat{A}}_{0},{\hat{A}}_{t}]{\hat{A}}_{1}\right)}^{2}=-2\det\left(\begin{array}{ccc} \mathrm{Tr}\;{\hat{A}}_{0}^{2} & \mathrm{Tr}\;{\hat{A}}_{0}{\hat{A}}_{t} & \mathrm{Tr}\;{\hat{A}}_{0}{\hat{A}}_{1}\\ \mathrm{Tr}\;{\hat{A}}_{t}{\hat{A}}_{0} & \mathrm{Tr}\;{\hat{A}}_{t}^{2} & \mathrm{Tr}\;{\hat{A}}_{t}{\hat{A}}_{1}\\ \mathrm{Tr}\;{\hat{A}}_{1}{\hat{A}}_{0} & \mathrm{Tr}\;{\hat{A}}_{1}{\hat{A}}_{t} & \mathrm{Tr}\;{\hat{A}}_{1}^{2} \end{array}\right).$$

The algebraic formula above can be used to determine a differential equation for $\hat{\zeta }(t)$ and its derivatives. Remember that, in (3.1), the matrices *A*_*i*_ are not traceless. Defining $\tau (t):=t^{\theta_{0}\theta_{t}/2}{\left(t-1\right)}^{\theta_{t}\theta_{1}/2}\hat{\tau }(t)$ it is straightforward to show that

3.11 $$t(t-1)\frac{d}{dt}\log\tau (t)=(t-1)\mathrm{Tr}\;A_{0}A_{t}+t\mathrm{Tr}\;A_{t}A_{1}=\hat{\zeta }(t)+\frac{(t-1)\theta_{0}\theta_{t}}{2}+\frac{t\theta_{1}\theta_{t}}{2}.$$

Then, using *A*_0_ + *A*_*t*_ + *A*_1_ = − *A*_∞_, equation (3.10), in terms of $\hat{\zeta }(t)$, becomes

3.12 $$\begin{array}{r} {\left(t(t-1){\hat{\zeta }}^{\prime \prime }(t)\right)}^{2}=-2\det\left(\begin{array}{ccc} \frac{\theta_{0}^{2}}{2} & t{\hat{\zeta }}^{\prime }-\hat{\zeta } & {\hat{\zeta }}^{\prime }+\frac{\theta_{0}^{2}+\theta_{t}^{2}+\theta_{1}^{2}-\theta_{\infty }^{2}}{4}\\ t{\hat{\zeta }}^{\prime }-\hat{\zeta } & \frac{\theta_{t}^{2}}{2} & (t-1){\hat{\zeta }}^{\prime }-\hat{\zeta }\\ {\hat{\zeta }}^{\prime }+\frac{\theta_{0}^{2}+\theta_{t}^{2}+\theta_{1}^{2}-\theta_{\infty }^{2}}{4} & (t-1){\hat{\zeta }}^{\prime }-\hat{\zeta } & \frac{\theta_{1}^{2}}{2} \end{array}\right). \end{array}$$

Equation (3.12) is known as the *σ*-form of the Painlevé VI equation (*σ*-PVI). Thus one can interpret the solution of (3.12), or, equivalently, of the Schlesinger equations (3.6), as representing a class of differential equations of the form (3.1)—and therefore of (3.3) whose solutions have the same monodromy parameters. The set is parametrized by the position of the singularity at *w* = *t*, and we will call it the isomonodromic deformation of the Heun equation.

The task is now to view the Heun equation (1.4) as an element of a family of an isomonodromically deformed system. It is clear from (3.3) and (3.4) that choosing

3.13 $$\lambda (t_{0})=t_{0},\;\mu (t_{0})=-\frac{K_{0}}{\theta_{t}},$$

one can arrive at the Heun equation in the form (1.4)—i.e. the equation without the extra singularity term at *w* = λ – as a smooth limit of the isomonodromic family. One can then think of these conditions as initial conditions for the Schlesinger equations, or, equivalently, for the Painlevé VI system. By adjusting the parameters so that *θ*_*t*_ = *θ*_*t*_0__ − 1 and *θ*_∞_ = *θ*_∞_0__ + 1, which implies that *q*_−_ *q*_+_ = *κ*_−_(1 + *κ*_+_) one recovers the exact form of (1.4) from (3.3).

When written in terms of the tau function, these conditions define a well-posed initial value problem for (3.12):

3.14 $$\begin{array}{rl} & {t(t-1)\frac{d}{dt}\log\tau (\theta_{i},\sigma_{ij},t)|}_{t=t_{0}}=t_{0}\frac{\theta_{t}\theta_{1}}{2}+(t_{0}-1)\frac{\theta_{0}\theta_{t}}{2}+t_{0}(t_{0}-1)K_{0},\\ & \;{\frac{d}{dt}\left[t(t-1)\frac{d}{dt}\log\tau (\theta_{i},\sigma_{ij},t)\right]|}_{t=t_{0}}=(\theta_{\infty }-\theta_{t})\frac{\theta_{t}}{2}, \end{array}$$

where the hat symbol has been dropped. The conditions above allow us to solve for the accessory parameters of (1.4) in terms of the monodromy data. These conditions along with the differential equation (3.12) guarantee at least one solution for the accessory parameters, due to general existence theorems for solutions.

Casting the accessory problem in terms of the tau function has more advantages. First, the tau function can be shown to be an analytic function of *t* except at the singular points *t* = 0, 1, ∞. It is a function of the invariant monodromy data, and its existence can be seen from (3.14) by standard theorems of existence of solutions to ODEs such as (3.12). The full set of arguments of *τ*, namely

$$\theta_{i}\in \{\theta_{0},\theta_{1},\theta_{t_{0}}-1,\theta_{\infty }+1\},\;\sigma_{ij}\in \{\sigma_{0t_{0}}-1,\sigma_{1t_{0}}-1,\sigma_{01}\},$$

can readily be computed for our problem using the method presented in §2. Our main motivation for framing the problem in terms of the tau function comes from the fact that asymptotic expansions for the latter in terms of the monodromy data can be computed [17]. Recently, the Painlevé VI tau function was related to Virasoro conformal blocks [26] and further connections to the partition function of supersymmetric gauge theories [21,22] allowed for a combinatorical construction of the full series [27]. Equations (3.14) are indeed generic and can be used for relating the monodromy data to the accessory parameters for any Heun differential equation. To our knowledge, the explicit relation (3.14) was cast for the first time in [18,19]. See [28] for a more recent discussion of the many different connections and applications.

Before delving into solutions to our particular conformal mapping problem, let us digress and consider an interpretation of (3.14). The first equation establishes the tau function as the generating function for the canonical transformation relating the accessory parameters to the monodromy data, whose phase space can be parametrized by *σ*_0*t*_, *σ*_1*t*_; see [29] for a suitable definition of Darboux coordinates in terms of monodromy data. The second condition can be understood from the *Toda equation* for tau functions [30]:

3.15 $$\frac{d}{dt}\left[t(t-1)\frac{d}{dt}\log\tau (t)\right]-\frac{(\theta_{\infty }-\theta_{t})\theta_{t}}{2}=c\frac{\tau^{+}(t)\tau^{-}(t)}{\tau^{2}(t)},$$

where c∈C is a *t*-independent constant; this establishes *t*_0_ as a zero of either *τ*^+^(*t*) or *τ*^−^(*t*) where *τ*^±^(*t*) are defined analogously to *τ*(*t*) but for systems with the modified monodromies

3.16 $$\theta_{i}^{\pm }=\{\theta_{0},\theta_{1},\theta_{t}\pm 1,\theta_{\infty }\mp 1\},\;\sigma_{ij}^{\pm }=\{\sigma_{0t}\pm 1,\sigma_{1t}\pm 1,\sigma_{01}\}.$$

The Toda equation can be obtained by direct construction from the Fuchsian system by multiplying the solution *Φ*(*w*) of (3.1) by diag((*w* − *t*)^ ± 1^, 1). These are known in the literature as Bäcklund or Schlesinger transformations. A tedious calculation shows that

$$\frac{d}{dt}\log\tau^{+}(t)=K+\frac{\lambda (\lambda -1)}{t(t-1)}\mu +\frac{\kappa_{-}(\lambda -t)}{t(t-1)}-\theta_{t}\left(\frac{1}{t}+\frac{1}{t-1}+\frac{1}{\lambda -t}\right).$$

Thus, *τ*^−^(*t*) is actually related to our Hamiltonian *K*, whereas *τ*^+^(*t*) is zero at *t* = *t*_0_, because of the condition λ(*t*_0_) = *t*_0_. The zeros of the isomonodronic tau function—the Malgrange divisor [31] are related to special points of the space of parameters where the RHp does *not* have a solution [32]. The fact that we encounter the accessory parameters literally at one step from insolvency surely has a deeper mathematical meaning and deserves further study. It is also worth noting that for any domain with a geometric interpretation, like the ones in the examples to follow, the corresponding *θ*_*i*_ will be real, and so will be the zero of the tau function 0 < *t*_0_ < 1, and the accessory parameter *K*_0_. We can pose the conjecture at this point that these are not only necessary but sufficient conditions, and leave the verification for future work.

## 4. Determination of accessory parameters

In view of the foregoing discussion, we propose a determination of the accessory parameters appearing in (1.4) from the equations

4.1 $$\tau^{+}(t_{0})=0,\;K_{0}=K(t_{0}),\;K(t):=\frac{d}{dt}\log\tau (\theta_{i},\sigma_{ij},t)-\frac{(\theta_{t_{0}}-1)\theta_{1}}{2(t-1)}-\frac{(\theta_{t_{0}}-1)\theta_{0}}{2t},$$

where explicit expansions for *τ*(*t*) near *t* = 0 and *t* = 1 are available from [27] and are recorded here in appendix A.

It is pointed out that the arguments—i.e. the monodromy data *ρ*—used in the tau function (4.1) are those used in the Fuchsian system:

$$\rho =\{\theta_{0},\theta_{t}=\theta_{t_{0}}-1,\theta_{1},\theta_{\infty }=\theta_{\infty_{0}}+1,\sigma_{0t}=\sigma_{0t_{0}}-1,\sigma_{1t}=\sigma_{1t_{0}}-1,\sigma_{01}\},$$

which in turn guarantees that the equation for the first line of *Φ*(*w*) (3.3) reduces to (1.4) when λ = *t*. On the other hand, the monodromy data used for *τ*^+^(*t*) is related to *ρ* by a shift

$$\rho^{+}=\{\theta_{0},\theta_{t_{0}},\theta_{1},\theta_{\infty_{0}},\sigma_{0t_{0}},\sigma_{1t_{0}},\sigma_{01}\},$$

being actually the monodromy parameters for the solutions of (1.4). For completeness we list the monodromy data for *τ*^−^(*t*)

$$\rho^{-}=\{\theta_{0},\theta_{t_{0}}-2,\theta_{1},\theta_{\infty_{0}}+2,\sigma_{0t_{0}}-2,\sigma_{1t_{0}}-2,\sigma_{01}\}.$$

From the numerical point of view, three ways of solving (4.1) are available.

(i) Numerical integration of the differential equation (3.12) satisfied by the tau function. The dependence of the solutions on monodromy data is computed from the asymptotic expressions given by Jimbo [17].
(ii) Algebraic evaluation of the Nekrasov sums (A 1). This is the method chosen for this article (even if it is not always the most computationally efficient). Even so, the method is found to give overall better results than an alternative numerical method due to Howell [15] and the convergence is fast for important examples. More significantly, it can yield an approximate analytical expression for relations satisfied by the required accessory parameters, as we show in §5.
(iii) Evaluation of the Fredholm determinant expression for the tau function given in [33]. This method has the advantages of the combinatorial expansion along with fast convergence. Examination of the efficacy of this method is the subject of ongoing work.

## 5. Illustrative calculations

Examples illustrating the new method are now presented. For comparison, and verification, we also give values of the accessory parameters obtained using Howell's method [15].

### (a) A generic polycircular arc domain

**Figure 2.**
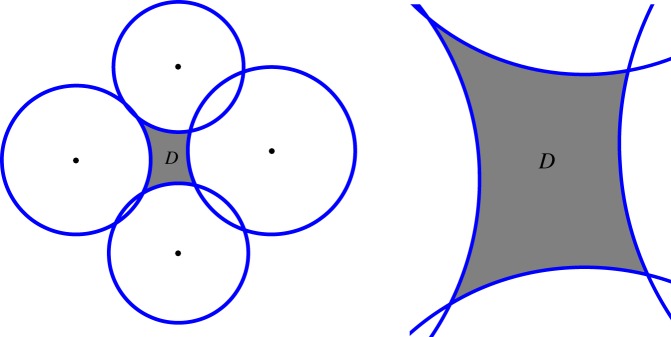
A generic polycircular arc domain *D* formed as the region enclosed by the circles centred at − 1.1, − i,  1 + 0.1i,  i with the respective radii 0.8, 0.75, 0.9, 0.7. (Online version in colour.)

The mapping from the upper half plane to the interior of the region displayed in figure 2 is now calculated. To implement the new method, we must first find the monodromy data according to (2.8) and (2.6). Results are recorded in table 1, reported correct to 10 digits. As stated above, the monodromy data consists of seven parameters *J*(*θ*_*i*_, *σ*_*ij*_) satisfying the Fricke–Jimbo relation (2.9), which should vanish up to numerical tolerance. The parameters *θ*_*i*_ correspond to the internal angles divided by *π* in figure 2. The composite monodromy parameter between consecutive pre-vertices, say 0 and *t*, may also be the angle between two arcs since $2\cos(\pi \sigma_{0t_{0}})=\mathrm{Tr}\;S_{1}{\bar{S}}_{4}S_{2}{\bar{S}}_{1}=\mathrm{Tr}\;S_{2}{\bar{S}}_{4}$, and therefore if the arcs *C*_2_ and *C*_4_ intersect, *πσ*_0*t*_0__ is the angle between them at the intersection. If they do not intersect, *σ*_0*t*_0__ will be a generic complex number.

**Table 1.** Monodromy data for example (a).

*θ*_0_	0.1827991846	*σ*_0*t*_0__	1 − 0.4304546489i
*θ*_*t*_0__	0.2869823004	*σ*_1*t*_0__	1 − 0.5385684561i
*θ*_1_	0.3673544015	*σ*_01_	0.9631297769 + 0.7221017400i
*θ*_∞_0__	0.0853271421	*J*(*θ*_*i*_, *σ*_*ij*_)	0

Using the monodromy data presented in table 1, the asymptotic expansion reviewed in appendix A is used to generate all relevant expressions in terms of tau functions. From the second equation of (3.14), it is clear that *t*_0_ is a zero of the following function:

$$L(t):=\frac{d}{dt}\left[t(t-1)\frac{d}{dt}\log\tau (t)\right]-\frac{(\theta_{\infty }-\theta_{t})\theta_{t}}{2}.$$

**Figure 3.**
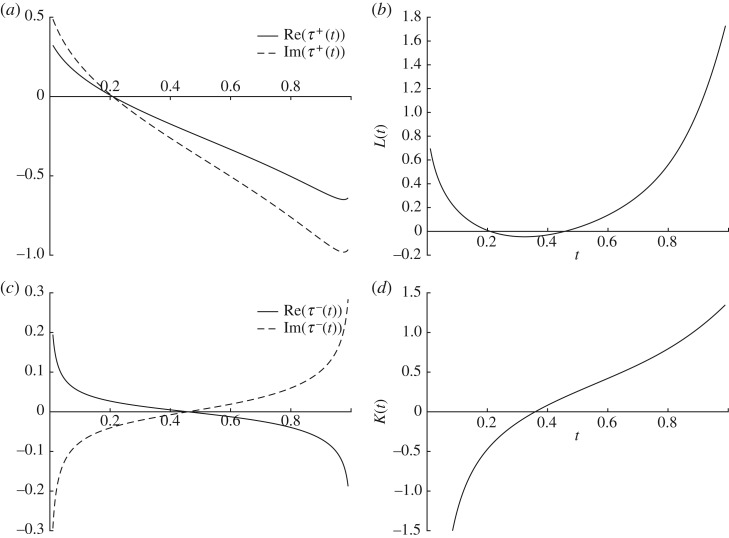
Plots of *τ*^+^(*t*) (*a*), *L*(*t*) (*b*), *τ*^−^(*t*) (*c*) and *K*(*t*) (*d*). The smallest zero of *L*(*t*) is a zero of *τ*^+^(*t*) while the larger one is a zero of *τ*^−^(*t*).

In fact, the zeros of *L*(*t*) come in pairs, each one corresponding to a zero of either *τ*^+^ or *τ*^−^, in agreement with the Toda equation (3.15). Figure 3 shows plots of these functions to illustrate the ‘factorization of the zeros’ of *L*(*t*).

The tau functions used here were generated using asymptotic expansions about *t* = 0 since *t*_0_ is found to be closer to 0 than to 1. Table 2 reports the accessory parameters *t*_0_ and *K*_0_ obtained by the new method to 10 digits of accuracy. Accessory parameters obtained from an implementation of the numerical scheme (based on a completely different construction) proposed by Howell [15] are also reported. (Note that results using Howell's method are also reported to 10 digits for comparison but only 4–6 digits of accuracy were expected.)

**Table 2.** Accessory parameters for example (a).

	new method	Howell's method
*K*_0_	−0.4364792362	−0.4365168488
*t*_0_	0.2086468690	0.2086251630

It should be emphasized that we are determining only the differential equation (1.1) satisfied by the mapping *f*(*w*). To determine *f*(*w*) completely, we must supplement (1.1) with (complex) initial conditions. Alternatively, we notice that if $\tilde{f}(w)$ satisfies (1.1), then so will the function *f*(*w*), related to $\tilde{f}(w)$ by a Möbius transformation

5.1 $$f(w)=\frac{a\tilde{f}(w)+b}{c\tilde{f}(w)+d},\;\left(\begin{array}{cc} a & b\\ c & d \end{array}\right)\in \text{SL}(2,C).$$

Hence one can simply guess initial conditions for the Schwarzian differential equation and find *a posteriori* a Möbius transformation that takes that solution to the one with the correct vertex positions and curvatures. This, as a rule, is the simplest part of the implementation. One only needs to pick an association $\tilde{f}(w_{i})\to f(w_{i})$ for three different points *w*_*i*_ to fix the transformation (5.1) and, therefore, determine *f*(*w*).

The desired zero is at *t*_0_≃0.209. However, there is actually more than one zero of *τ*^+^ in the interval (0, 1): to within the accuracy of our numerical method, we identify a second zero close to *t* = 0 at *t*_0_≃1.0706 × 10^−7^. The *t*_0_ and *K*_0_ extracted from this zero yield an ‘isomonodromic’ region in which the image of the real line follows one of the circles that make up the boundary of the region once before continuing on to the next piece of the boundary. The zero of interest is the one that yields a boundary that is free of self-intersections. One notes that the additional zeros should indeed occur near 0 due to the interpretation of *t*_0_ as the anharmonic ratio between the positions of the singular points.

In our numerical tests, we noticed a greater discrepancy between the results of Howell's numerical procedure and those generated by the new method when *t*_0_ is very close to either 0 or 1. This is due to the well-known crowding phenomenon associated with the traditional approaches to solving for the accessory parameters in conformal mapping problems. In the new method introduced here, this problem is bypassed yielding more accurate solutions easily. Indeed, since the tau function expansion converges faster in such circumstances, it is even *desirable* (for our method) that *t*_0_ is near to one of the critical points. We explore ramifications of this observation again in example §5(c) to follow.

### (b) A circular meniscus spanning a rectangular groove

**Figure 4.**
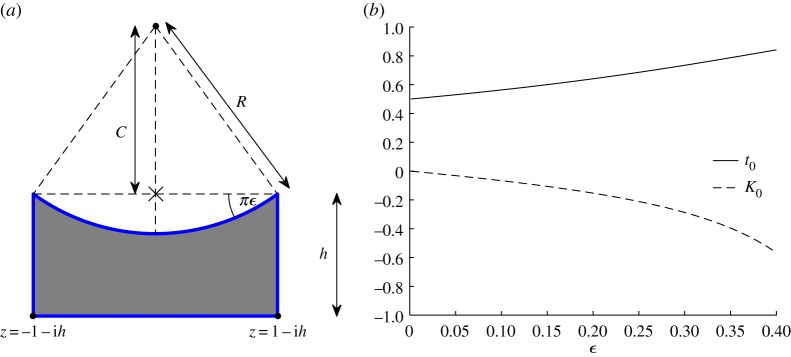
(*a*) Schematic of a meniscus on the top of a rectangular groove. Cross symbol ( × ) indicates the origin. Geometric arguments show that *C* = *R*cos*πϵ* and R=csc⁡πϵ. (*b*) Plot of the accessory parameters as functions of *ϵ* when *h* = 2. (Online version in colour.)

This example involves a circular meniscus forming the upper side of a rectangular groove as shown in figure 4. When *h* → ∞, so that the two lower vertices merge at infinity, this geometry can be described by a conformal mapping that is a hypergeometric function. Such a mapping has been found by Morris [34] and used by him in a heat transfer problem involving an evaporating meniscus. The following construction of the mapping for *h* < ∞ should be of use in generalizing his analysis to finite-depth grooves.

The Schwarz functions for the separate boundary portions shown in figure 4 are as follows. On the bottom straight line edge, we have $\bar{z}=z+2ih$; on the left- and right-hand straight line edges, we have $\bar{z}=\pm 2-z$. For a given *ϵ* we find, from simple trigonometry, that

$$\frac{1}{R}=\sin\pi \epsilon ,\;C=R\cos\pi \epsilon =\cot\pi \epsilon .$$

Hence the upper circular arc is given by |*z* − i*C*|^2^ = *R*^2^ or

$$\bar{z}=-iC+\frac{R^{2}}{z-iC}=-i\cot\pi \epsilon +\frac{{\mathrm{cosec}}^{2}\pi \epsilon }{z-i\cot\pi \epsilon }.$$

From these Schwarz functions, the monodromy matrices can easily be determined following the prescription given in §2.

To calculate *t*_0_, we look for the zero of *τ*^+^(*t*) that is closest to the midpoint of the interval (0,1). Depending on whether the zero is in the first half of the interval—just a few terms is enough to determine that—we may, in general, choose to use the expansion about 0 or 1 to speed up the evaluation of the tau function: here we find *t*_0_ falls in the interval (1/2,1) as can be seen from the plot in figure 4*b*. However, it should be noted that we are not able to make use of the expansion of the tau function (A 1) around *t* = 1,^1^ due to fact that (A 1) presupposes that the monodromy parameters satisfy the ‘generic conditions’ [17,33]:

5.2 $$\sigma_{0t_{0}}\notin Z,\;\theta_{0}\pm \theta_{t_{0}}\pm \sigma_{0t_{0}}\notin 2Z,\;\theta_{1}\pm \theta_{\infty_{0}}\pm \sigma_{0t_{0}}\notin 2Z.$$

The first condition seems a technical point on the poles and zeros of the structure constants (see (A 2)), whereas the last two conditions are related to the reducibility of the monodromy group, because their violation is equivalent to the commutativity between the corresponding single-point monodromy matrices. If any of these is violated, the tau function has to be computed through a limiting procedure. In this particular example, when we make the exchange 0↔1 in the indices of the monodromy parameters in the relations above, and at least one of the conditions (5.2) is not satisfied, and thus the expansion around *t* = 1 is not defined.

This leads to the following question: if only one tau function expansion is available and *t*_0_ is far from the point about which the expansion is performed, what is the best way to proceed? Three possibilities are as follows. (i) A large number of terms in the available expansion can be computed to produce accessory parameters of the desired accuracy. This can be computationally expensive. (ii) The first few terms of the expansion are used to generate initial conditions for the differential equation (3.12) (close to the expansion point) and then the differential equation is integrated until the condition *L*(*t*) = 0 is satisfied to some numerical tolerance. Of course, some problems may arise since *L*(*t*) may in general have more than one zero, but one can always use a truncated *τ*^+^(*t*) expansion to quickly distinguish the correct *t*_0_. We have found that this approach, using the differential equation in tandem with the tau function expansion, is faster for some configurations. (iii) We can use a perturbative approach based on altering the curvature of one (or more) of the sides and taking a limit. This is explored in detail in example 5(d).

### (c) Semi-circular obstacle in an infinite channel

**Figure 5.**
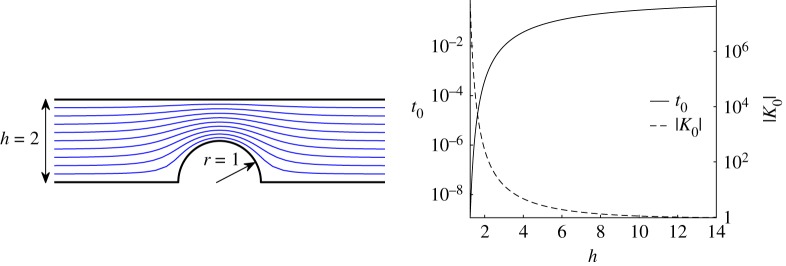
Streamlines for potential flow over a semicircular obstacle, of unit radius, in a channel of height *h* = 2. The accessory parameters are found to be *t*_0_ = 3.904625 × 10^−4^ and *K*_0_ = − 2.725462 × 10^2^. Graphs of the accessory parameters as functions of channel height *h* are also shown (here *K*_0_(*h*) < 0 and |*K*_0_| is plotted). (Online version in colour.)

Unbounded domains are also amenable to our approach. Consider the problem of finding the streamlines of uniform potential flow over a semicircular obstacle in an infinite channel (figure 5). This geometry is ubiquitous in applications, and several authors have considered the matter of constructing a conformal mapping to this ‘disc-in-channel’ geometry [35–37]. Given the relevant uniformizing map, the complex potential and hence the streamlines, follow immediately on use of standard potential theory methods.

The simple and composite monodromy data associated with this domain are *θ*_0_ = *θ*_∞_0__ = 0, *θ*_*t*_0__ = *θ*_1_ = 0.5 with *σ*_0*t*_ = *π*^−1^cos^−1^( − *h*), *σ*_1*t*_ = 0 and *σ*_01_ = *π*^−1^cos^−1^(*h*). Figure 5 shows *t*_0_ and *K*_0_ as functions of the channel width *h* (for fixed obstacle radius). Again, we only have available a tau function expansion about *t* = 0, but, in contrast to the previous example, this presents no practical problem because *t*_0_ is close to zero. For the same reason, just a few terms in the tau function expansion are enough to find accurate approximations to *t*_0_ and *K*_0_.

It should be noted that direct methods of integration based on (1.1) in such highly elongated regions are known to be subject to numerical inaccuracies (which can be mitigated, for example, by introducing an intermediate transformation to a ‘strip’ domain [15]). Such complications are avoided by our new approach. Moreover, if *t*_0_ is close to one of the singular points at 0 or 1 this can be of great advantage in our approach in that only a few terms (often only the first term) in the expansion of the tau function are needed. For instance, let us fix *h* = 2. It turns out we can neglect all the terms in the expansion for the tau function coming from the conformal blocks except for the first $B_{\emptyset ,\emptyset }=1$, then use only the two most contributing terms in the expansion and still produce good approximations. To lowest order in *t*_0_, the relevant terms of *τ*^+^(*t*) comprise only the *n* = 0, − 1 terms appearing in (A 1) and, for each *n*, only the coefficients in B depending on the Young diagrams of zero length. A simple calculation shows that the zero of *τ*^+^(*t*) occurs at

$$t_{0}^{1-\sigma }≃\frac{1+\sin(\pi \sigma )}{1-\sin(\pi \sigma )}\frac{\Gamma^{4}(1/4+(1/2)\sigma )}{\Gamma^{4}(5/4-(1/2)\sigma )}\frac{\Gamma^{2}(1-\sigma )}{\Gamma^{2}(\sigma -1)},\;h=-\cos(\pi \sigma ),$$

where *σ* = *σ*_0*t*_0__ and *h* is the height of the channel. Using this approximation, the zero of *τ*^+^(*t*) for *h* = 2 is *t*_0_≃3.905353 × 10^−4^. To approximate *K*_0_, it is sufficient to retain only one term in the expansion of *τ*(*t*) yielding

$$K_{0}={\frac{d}{dt}\log\tau (\theta_{i},\sigma_{ij},t)|}_{t=t_{0}}-\frac{(\theta_{t_{0}}-1)\theta_{1}}{2(t_{0}-1)}-\frac{(\theta_{t_{0}}-1)\theta_{0}}{2t_{0}}≃\frac{{\left(\sigma -1\right)}^{2}-{\left(\theta_{0}+\theta_{t_{0}}-1\right)}^{2}}{4t_{0}}.$$

For *h* = 2, *K*_0_≃ − 2.725292 × 10^2^.

This is evidence that, for certain geometries, the new method can be very simple to apply *and* allows the accessory parameters to be determined as zeros of simple analytical expressions. Remarkably, these particular instances arise precisely when the usual numerical conformal mapping constructions face difficulties due to the well-known crowding phenomenon.

### (d) The Schwarz–Christoffel mapping to a rectangle

All the examples so far have involved ‘circular-arc’ polygons where at least one side of the quadrilateral has non-zero curvature. A polygon, whose sides are all straight lines (zero curvature), is a special case and the conformal mapping can be constructed using the classical SC formula [8,13]. In the theory of SC mapping, it is not usual to even consider second-order Fuchsian differential equations. We now show, however, that there is significant advantage in doing so and approaching the case of a polygon as a ‘zero curvature limit’.

Consider the conformal mapping to the interior of a rectangle. It can be shown that the matrices *S*_*i*_ associated with straight sides are lower triangular, which in turn implies that all monodromy matrices have the same form. Moreover, the elements in the diagonal of *M*_*i*_, the only ones which contribute to the monodromy data in this case, do not depend on the aspect ratio of the rectangle. Thus, the association $\rho \to \{t_{0},K_{0}\}$ is spoiled since *t*_0_, at least, must depend on the aspect ratio.^2^ Therefore, in the case of polygons, the new method cannot be applied directly.

**Figure 6.**
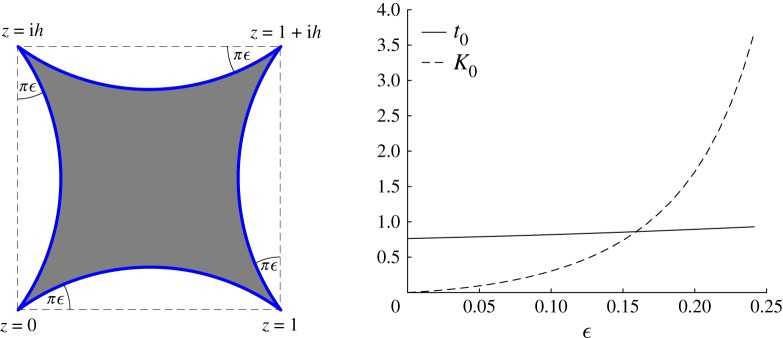
A ‘deformed’ rectangle where the sides are replaced by circular arcs making angle *πϵ* with the undeformed straight sides. Also shown are graphs of *t*_0_(*ϵ*) and *K*_0_(*ϵ*) for the ‘deformed’ rectangle (with *h* = 1.3) as a function of *ϵ*. (Online version in colour.)

However, we have found that a small curvature perturbative approach can produce the required values of the SC accessory parameters. The key idea of this small curvature perturbation is illustrated in figure 6. When we make *ϵ* = 1 × 10^−12^, where *ϵ* measures the deformation from zero curvature, the new method relates the aspect ratio *h* to *t*_0_ in excellent agreement with that produced using the usual SC theory (which leads to a formula for the relationship between these parameters [7] using elliptic integrals). In addition, numerical investigations regarding the new method allowed for the discovery of a special class of conformal mappings having the same accessory parameters: *t*_0_ = 0.5 and *K*_0_ = 0. They represent quadrilaterals illustrated by figure 6, with *h* = 1 and $0<\epsilon \leq \frac{1}{4}$. Notice that when $\epsilon =\frac{1}{4}$, all internal angles of the target domain vanish.

This evidence also motivates the conjecture: the zero curvature limits of *t*_0_(*ϵ*) and *K*_0_(*ϵ*) as ϵ→0 exist and precisely determine the accessory parameters associated with the rectangle. A more general conjecture (for any polygon) is expected to hold.

This novel determination of the accessory parameter for SC mappings—which is quite distinct from any extant approaches—deserves more careful investigation. In terms of monodromy, SC domains are characterized by the additive property of the monodromies *σ*_*ij*_ = *θ*_*i*_ + *θ*_*j*_, along with Fuchs relation $\sum_{i}\theta_{i}=0$. The particular fact that *σ*_0*t*_ = *θ*_0_ + *θ*_*t*_ means that (i) the *s* parameter (A 4) involved in the tau function expansion (A 1) diverges and (ii) there are poles in the Barnes function in (A 2). A careful limit can be taken yielding a finite result for the tau function—see, for instance, eqn (1.9) in [17]. The limit can be compared with known results for the case of rectangles, where it is established for some time that the accessory parameter *t*_0_ is given in terms of a ratio of elliptic functions [38]. Presumably, these are related to the Picard family of solutions for the tau function [39]. This zero curvature limit of the tau function is the subject of ongoing work.

We finish by pointing out that, in examples 5(b) and (c), slight deformations of the straight sides could have been used to overcome the potential difficulty associated with the lack of availability of an expansion of the tau function about one of the singular points.

## 6. Discussion

It is well known that the uniformization map for triangles formed with geodesic arcs in the Lobachevsky plane can be written in terms of ratio of hypergeometric functions, or ‘triangle functions’. The corresponding map for circular arc quadrilaterals can be written as a ratio of solutions of the Heun equation. However, unlike the hypergeometric case, the relevant Heun equation itself depends not only on the internal angles but also on two additional parameters, the accessory parameters *t*_0_ and *K*_0_. This paper has shown how to use the isomonodromic tau function associated with the Painlevé VI equation to determine these two parameters.

This was done by considering the Riemann–Hilbert problem (RHp) of finding the ODE associated with a function having prescribed singular behaviour. For the generic polycircular arc domain, we showed—using the Schwarz reflection principle—that, given a target geometry, we are able to determine all monodromy transformations—realized in our case by square two-dimensional matrices obtained by consideration of the local Schwarz function associated with each boundary arc. For the case of four sides, we then associated the monodromy data with an isomonodromic tau function introduced by Miwa, Jimbo and Ueno [16,17] from which the accessory parameters can be deduced by imposing conditions (4.1).

The proposed expansions for the tau function [26] were then used to extract the accessory parameters. We found not only very good agreement with other methods, but also that the analytic expansion for the tau function provides better accuracy with the same, or less, computational effort. Situations where the accessory parameter *t*_0_ comes close to 0 or 1 are particularly well suited to the analytical approach, due to the fast convergence of the expansions and the absence of ‘crowding’ problems that affect other numerical approaches. We find excellent numerical accuracy with relatively small computational effort for a variety of quadrilaterals, including ones with straight lines as edges, as well as unbounded domains.

Standard techniques for numerical solution of the accessory parameter problem for SC mappings [13] or mappings to circular arc polygons [6,10] invariably rely on enforcing geometrical conditions associated with the target domain. This usually requires some form of nonlinear iteration on the accessory parameters until those geometrical constraints are met. The new approach advocated here offers the unique perspective of relying not on geometrical conditions but on a completely different set of mathematical constraints associated with isomonodromic deformations.

The problem of determining the accessory parameters when the number of vertices is greater than four naturally arises. Isomonodromic tau functions exist for a general number of monodromies, so the suggestion that this could form a viable route to finding the corresponding uniformization map for generic polycircular arc domains now seems reasonable.

Another compelling course of action is the study of similar mappings on higher genus Riemann surfaces, as relevant to multiply connected polycircular domains, for example [8,11].

Other questions arise from the numerical observations made here. We saw that the tau function has a single zero on the interval (0, 1) associated with the accessory parameters for the ‘fundamental domain’ without self-intersections. It would be interesting to know if this uniqueness—up to global Möbius transformations—is indeed a feature of uniformizing maps of regions having a geometric interpretation. The zero curvature limit discussed here in the application to the SC map can yield an analytical solution for the accessory parameters—it seems to be related to the Picard solutions for Painlevé VI—but a proof of this is currently lacking.

A deeper mystery concerns trying to reconcile conditions (4.1) with previous work on the isomonodromic tau function and its relation to the RHp. Previously, it had been folklore that the zeros of the tau function are related to points in parameter space where the RHp does not have a solution. This can be understood from the fact that zeros of the tau function are simple, and hence the accessory parameters, which are given by the logarithmic derivative of the tau function, should not be defined there. Surprisingly, the accessory parameter of the ODE sought is related to this one by a Schlesinger transformation. This matter merits further investigation.

## Figures and Tables

**Figure 7.**
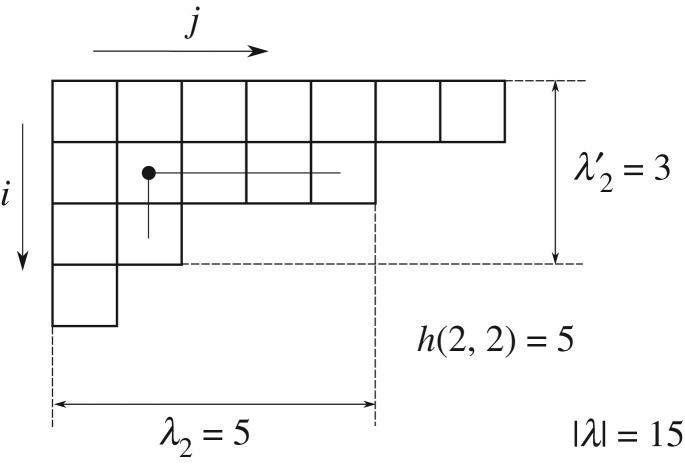
A Young diagram representation for the partition λ = {7, 5, 2, 1}. λ_2_ is the number of boxes in the second row, λ′_2_ is the number of boxes in the second column and the hook length of the box (2, 2) is the number of elements in the second column bellow that box plus the number of boxes to the right of it, in its row, plus one, to account for itself. |λ| is the size of the diagram.

## Notes

1 As explained in appendix A, we could obtain an expansion about *t* = 1 by permuting the vertices and the pre-vertices. In this case, *t*_0_ would fall in the interval (0,1/2) and an expansion about *t* = 0 would then not be possible.

2 *K*_0_ = 0 for any usual polygon with four sides. The corresponding tau function also yields this value as the contributions from the conformal blocks in the case *σ*_*ij*_ = *θ*_*i*_ + *θ*_*j*_ is zero (F. Novaes 2016, unpublished data).

## Data accessibility

This article has no additional data.

## Appendix A. Asymptotic expansion for the Painlevé VI tau function

A formula for the Painlevé VI tau function expansion was proposed in [26,27], building from the AGT conjecture. In [40,41], it was shown that the asymptotic formula does satisfy the Painlevé VI differential equation (3.12). Whether every solution of (3.12) allows for such an expansion is still an open question. The structure comes from equating the Painlevé VI tau function to an expansion in terms of conformal blocks of a certain correlation function in conformal field theory:

A 1 $$\tau (t)=\sum_{n\in Z}C(\theta_{0},\theta_{t},\theta_{1},\theta_{\infty },\sigma_{0t}+2n)s^{n}t^{({\left(\sigma_{0t}+2n\right)}^{2}-\theta_{0}^{2}-\theta_{t}^{2})/4}B(\theta_{0},\theta_{t},\theta_{1},\theta_{\infty },\sigma_{0t}+2n;t).$$

The details are outlined in [26]. Indeed, the equation above expresses the tau function as an asymptotic expansion around *t* = 0. The action of the braid group on the singular points in the Fuchsian system allows for similar expansions around *t* = 1 and *t* = ∞ [17]. The structure constants *C* in (A 1) can be written as

A 2 $$C(\theta_{0},\theta_{t},\theta_{1},\theta_{\infty },\sigma )=\frac{\prod_{\alpha ,\beta ,=\pm }G(1+(1/2)(\theta_{1}+\alpha \theta_{\infty }+\beta \sigma ))G(1+(1/2)(\theta_{t}+\alpha \theta_{0}+\beta \sigma ))}{\prod_{\alpha =\pm }G(1+\alpha \sigma )},$$

where the classical Barnes function *G*(*z*) satisfies the functional equation *G*(1 + *z*) = *Γ*(*z*)*G*(*z*) and can be defined according to

A 3 $$G(1+z)={\left(2\pi \right)}^{z/2}\exp\int_{0}^{\infty }\frac{dt}{t}\left[\frac{1-e^{-zt}}{4\sinh^{2}(t/2)}-\frac{z}{t}+\frac{z^{2}}{2}e^{-t}\right],\;\text{Re }z>-1.$$

Moreover, *s* can be calculated in terms of the monodromy data as

A 4 $$s=\frac{\left(w_{1t}-2p_{1t}-p_{0t}p_{01})-(w_{01}-2p_{01}-p_{0t}p_{1t})\exp(\pi i\sigma_{0t}\right)}{\left(2\cos\pi (\theta_{t}-\sigma_{0t})-p_{0})(2\cos\pi (\theta_{1}-\sigma_{0t})-p_{\infty }\right)},$$

where *p*_*i*_ = 2cos*πθ*_*i*_,  *p*_*ij*_ = 2cos*πσ*_*ij*_,  *w*_1*t*_ = *p*_1_*p*_*t*_ + *p*_0_*p*_∞_ and *w*_01_ = *p*_0_*p*_1_ + *p*_*t*_*p*_∞_. Because of the Fricke–Jimbo relation (2.9), the monodromy parameters are not all independent.

The last term in (A 1), $B(\theta_{0},\theta_{t},\theta_{1},\theta_{\infty },\sigma_{0t};t)$, corresponds in conformal field theory to the conformal block function $F_{c=1}(\frac{1}{4}\theta_{0}^{2},\frac{1}{4}\theta_{t}^{2},\frac{1}{4}\theta_{1}^{2},\frac{1}{4}\theta_{\infty }^{2},\frac{1}{4}\sigma_{0t}^{2};t)$ where $\frac{1}{4}\theta_{i}^{2}$ represent the conformal dimensions of the fields in a four-point correlation function, $\frac{1}{4}\sigma_{0t}^{2}$ stands for the intermediate conformal dimension, and *c* = 1 is the central charge. By the AGT relation, conformal blocks can be expanded in terms of Nekrasov functions, which implies

A 5 $$B(\theta_{0},\theta_{t},\theta_{1},\theta_{\infty },\sigma ;t)={\left(1-t\right)}^{\theta_{t}\theta_{1}/2}\sum_{\lambda ,\mu \in Y}B_{\lambda ,\mu }(\theta_{0},\theta_{t},\theta_{1},\theta_{\infty },\sigma )t^{|\lambda |+|\mu |},$$

where the sum is over the Young diagrams λ and *μ* contained in Y, the set of all such diagrams which represent ordered partitions of integers.^3^ So, for instance, since 15 = 7 + 5 + 2 + 1, one possible partition for the integer 15 can be represented as λ = {7, 5, 2, 1}, or by the Young diagram in figure 7. The size of the diagram is given by the number of boxes in it, thus |λ| = 15. Furthermore,

A 6 $$\begin{array}{rl} B_{\lambda ,\mu }(\theta_{0},\theta_{t},\theta_{1},\theta_{\infty },\sigma ) & =\prod_{(i,j)\in \lambda }\frac{\left({\left(\theta_{t}+\sigma +2(i-j)\right)}^{2}-\theta_{0}^{2})({\left(\theta_{1}+\sigma +2(i-j)\right)}^{2}-\theta_{\infty }^{2}\right)}{16h_{\lambda }^{2}(i,j){\left(\lambda_{j}^{\prime }+\mu_{i}-i-j+1+\sigma \right)}^{2}}\\ & \;\times \prod_{(i,j)\in \mu }\frac{\left({\left(\theta_{t}-\sigma +2(i-j)\right)}^{2}-\theta_{0}^{2})({\left(\theta_{1}-\sigma +2(i-j)\right)}^{2}-\theta_{\infty }^{2}\right)}{16h_{\mu }^{2}(i,j){\left(\lambda_{i}+\mu_{j}^{\prime }-i-j+1-\sigma \right)}^{2}}, \end{array}$$

where (*i*, *j*) denotes the coordinates of the boxes in the tableau, λ_*i*_ stands for the number of boxes in the row *i*, from the top to the bottom of the diagram λ, λ′_*j*_ is the number of boxes in the column *j*, and *h*_λ_(*i*, *j*) = λ_*i*_ − *i* + λ′_*j*_ − *j* + 1 is called the hook length. For instance, suppose we want to calculate the first few contributions to B:

A 7

We assign coordinates (*i*, *j*) to each box in the Young diagrams λ, *μ* in $B_{\lambda ,\mu }$ according to figure 7 and then calculate the coefficients in the series above by using (A 6). The symbol ∅ stands for ‘partition of zero’, and the product over the coordinates of ∅ in (A 6) equals 1, by convention, so that $B_{\emptyset ,\emptyset }=1$. In fact, the first few terms in this asymptotic expansion were found by Jimbo [17] who also showed that asymptotic expansions around the other critical points *t* = 1, ∞ are analogous to the one around *t* = 0. To produce accurate results more efficiently when *t*_0_≲1, it may be convenient, although not strictly necessary, to use the asymptotic expansion around *t* = 1 which is obtained when one makes the following interchanges in (A 1):

A 8 $$t↔1-t,\;\theta_{0}↔\theta_{1},\;\sigma_{0t}↔\sigma_{1t}$$

and, in the definition of *s*, one must change the exponential term as $\exp(\pi i\sigma_{0t})\to \exp(-\pi i\sigma_{1t})$. Another approach to deal with the case with *t*_0_≲1 is to make a cyclic change in the association between the vertices and the pre-vertices until 0 < *t*_0_ ≤ 0.5.
